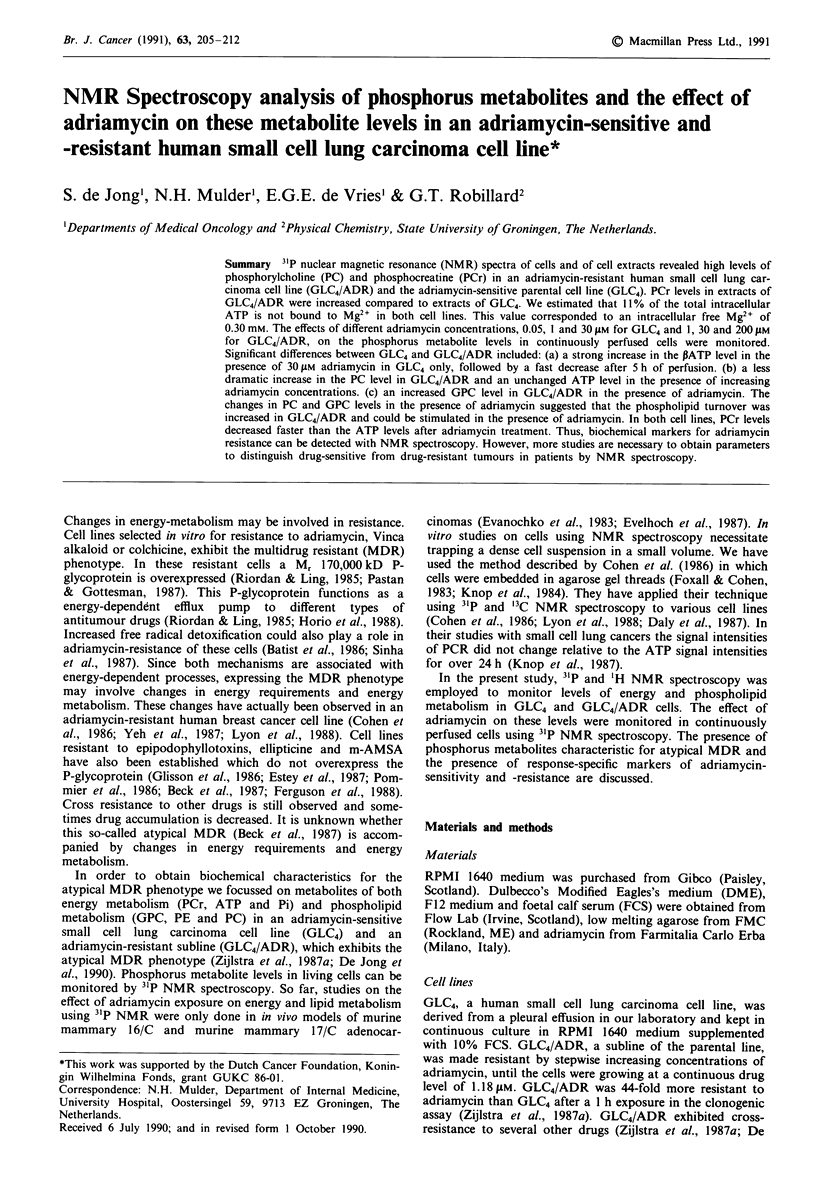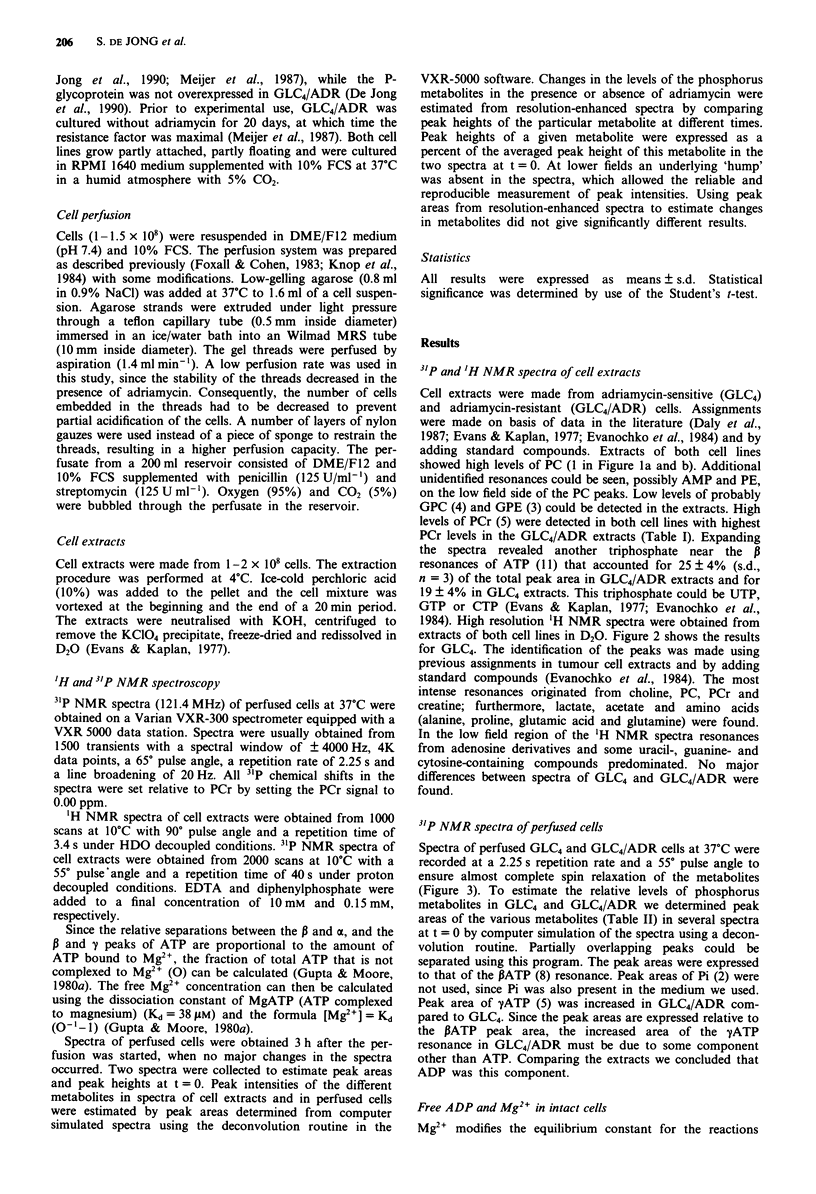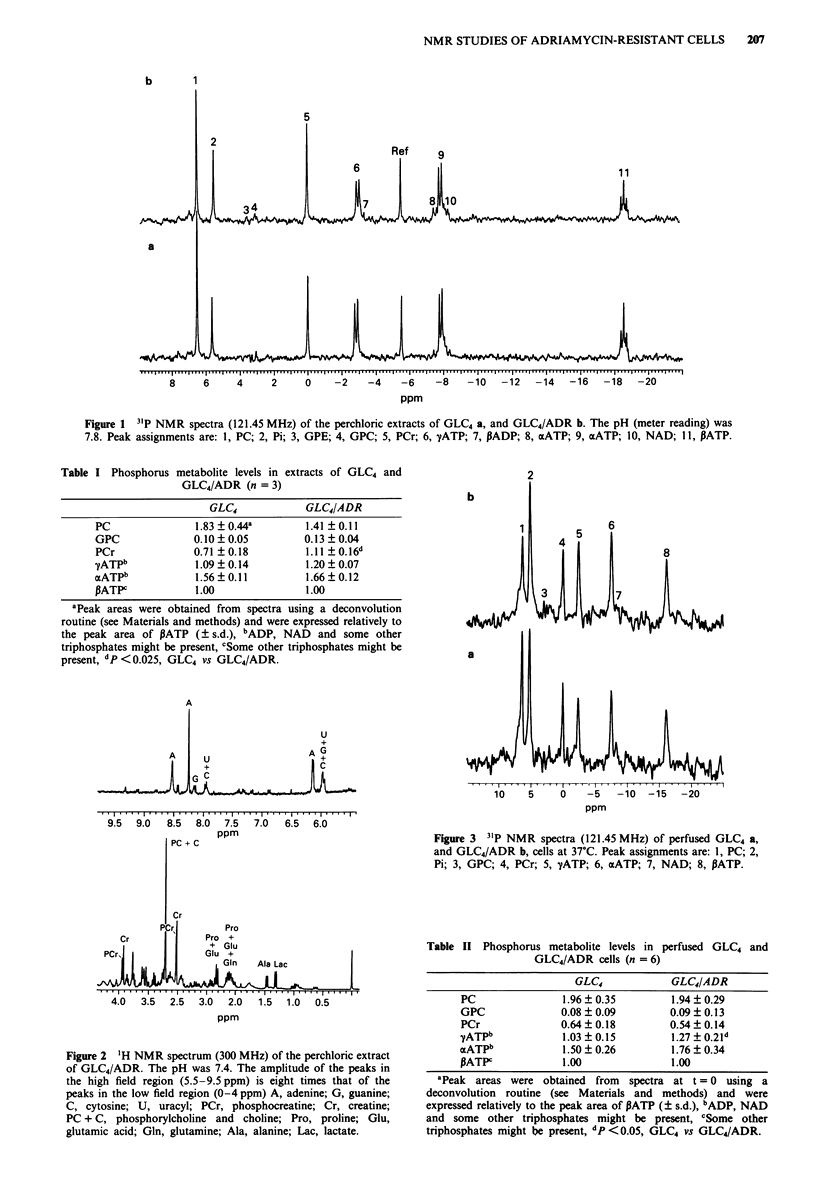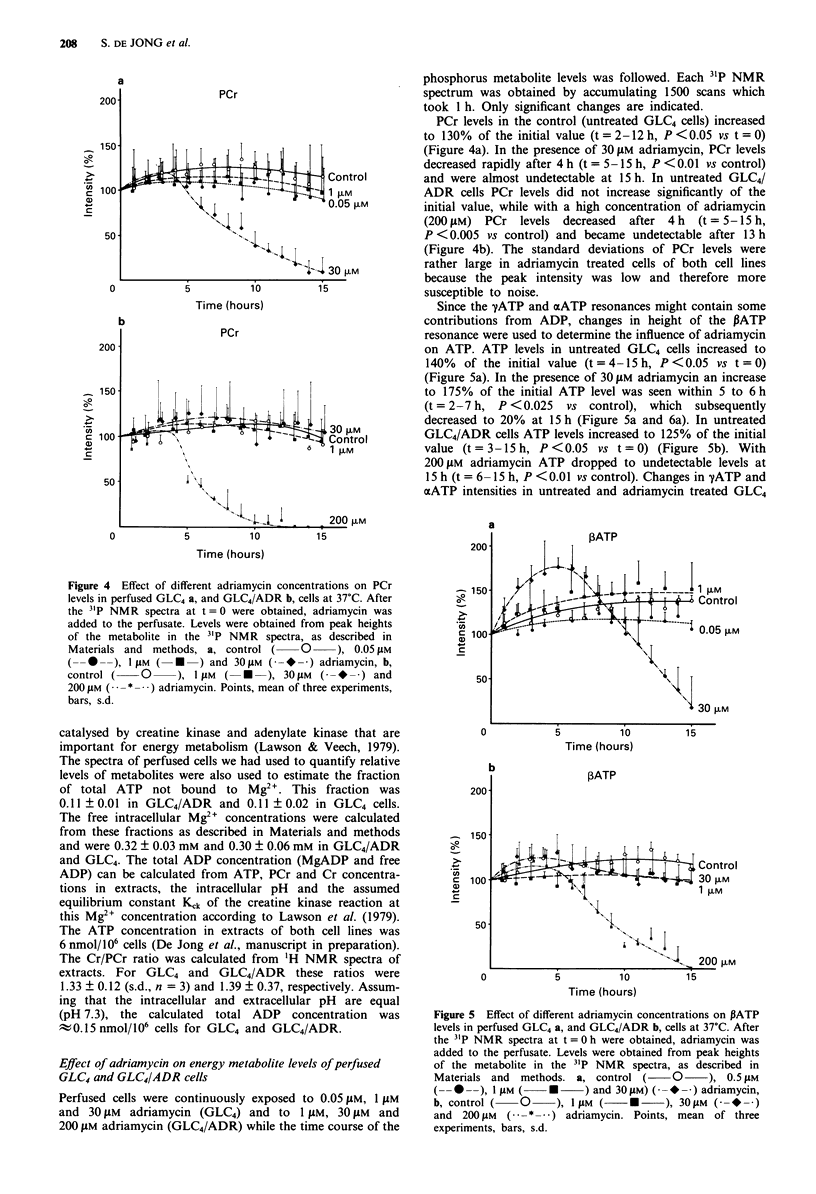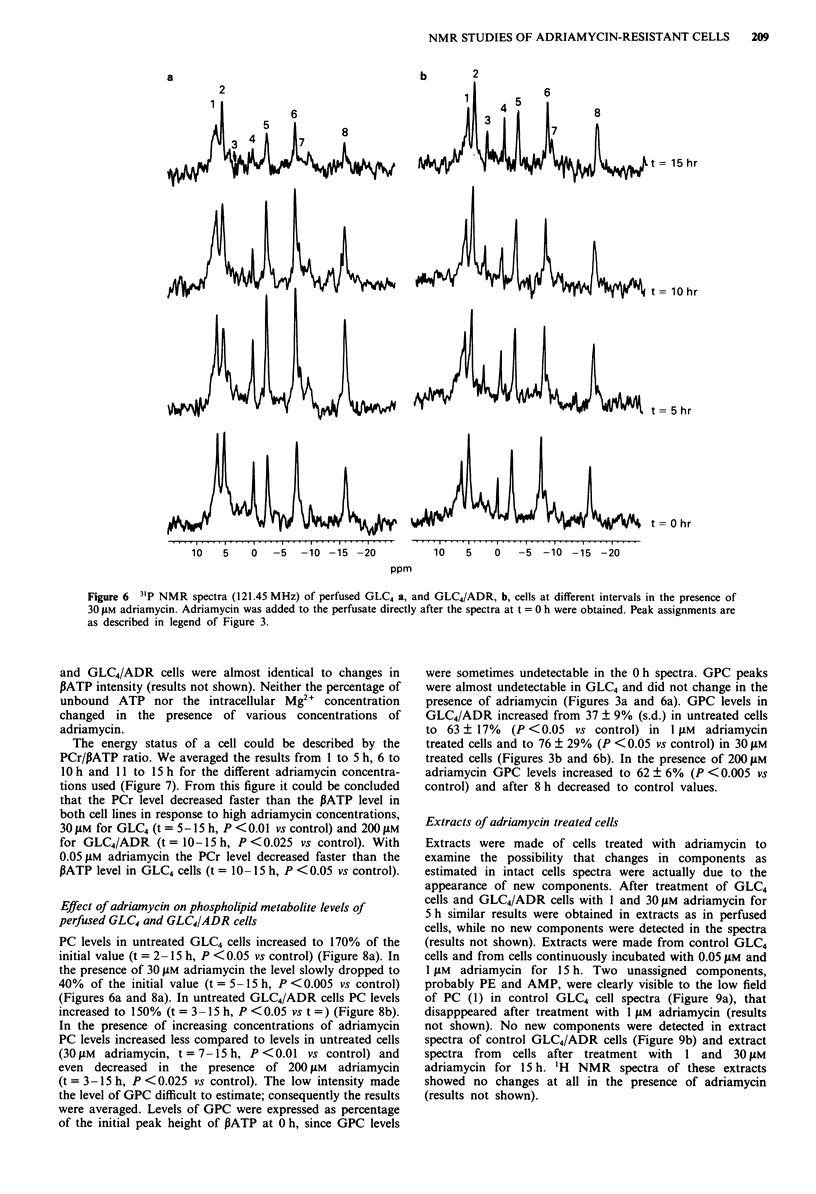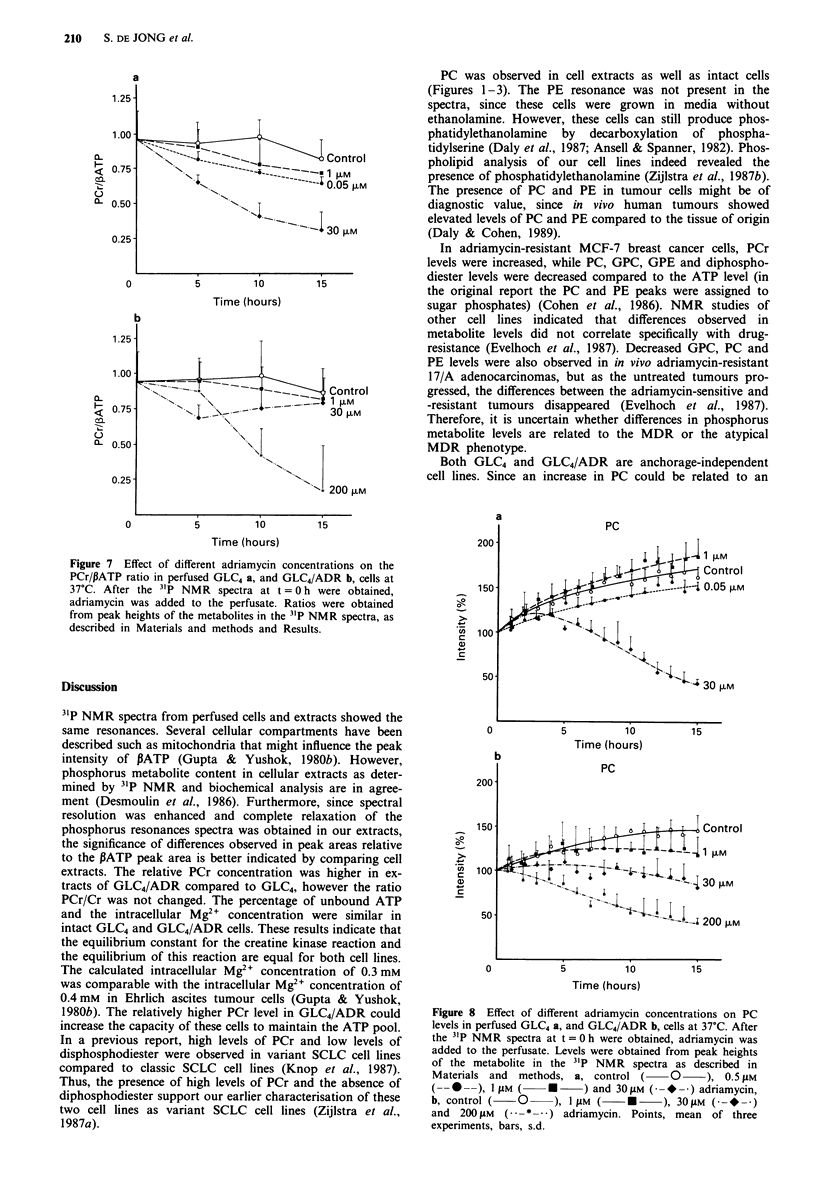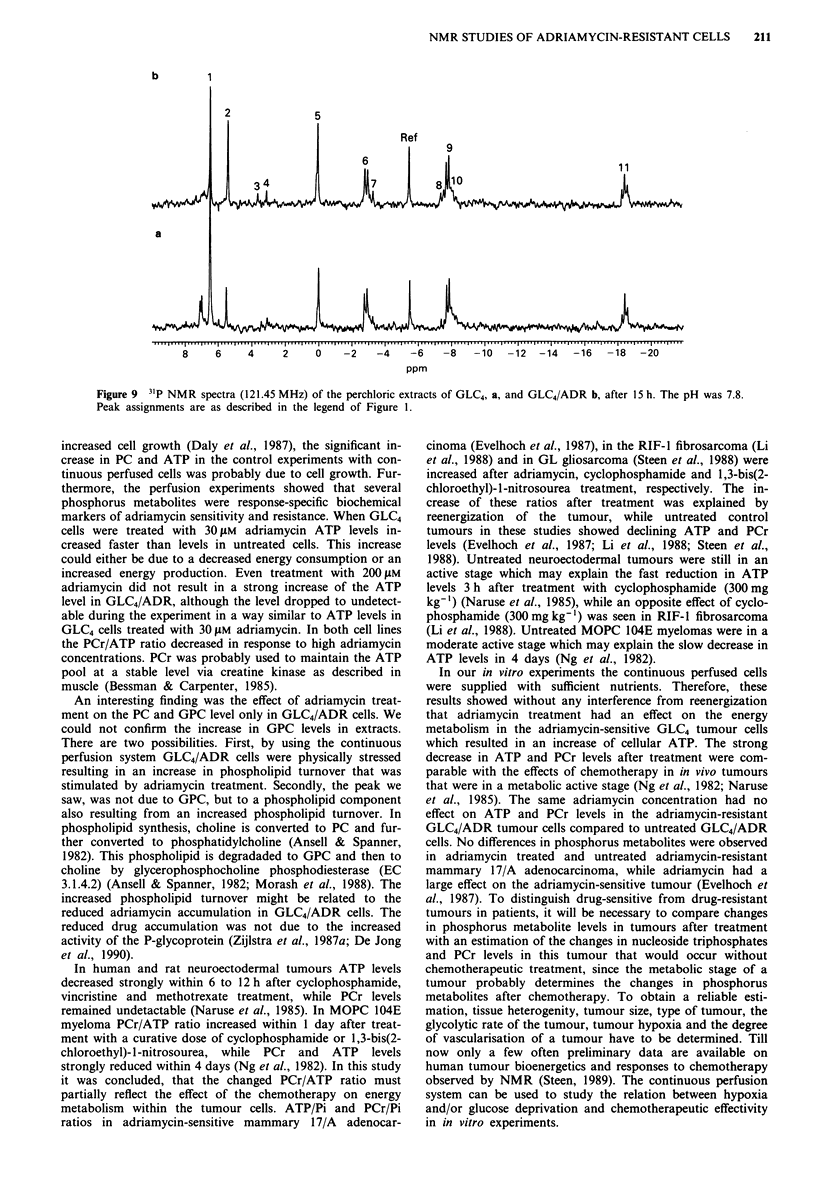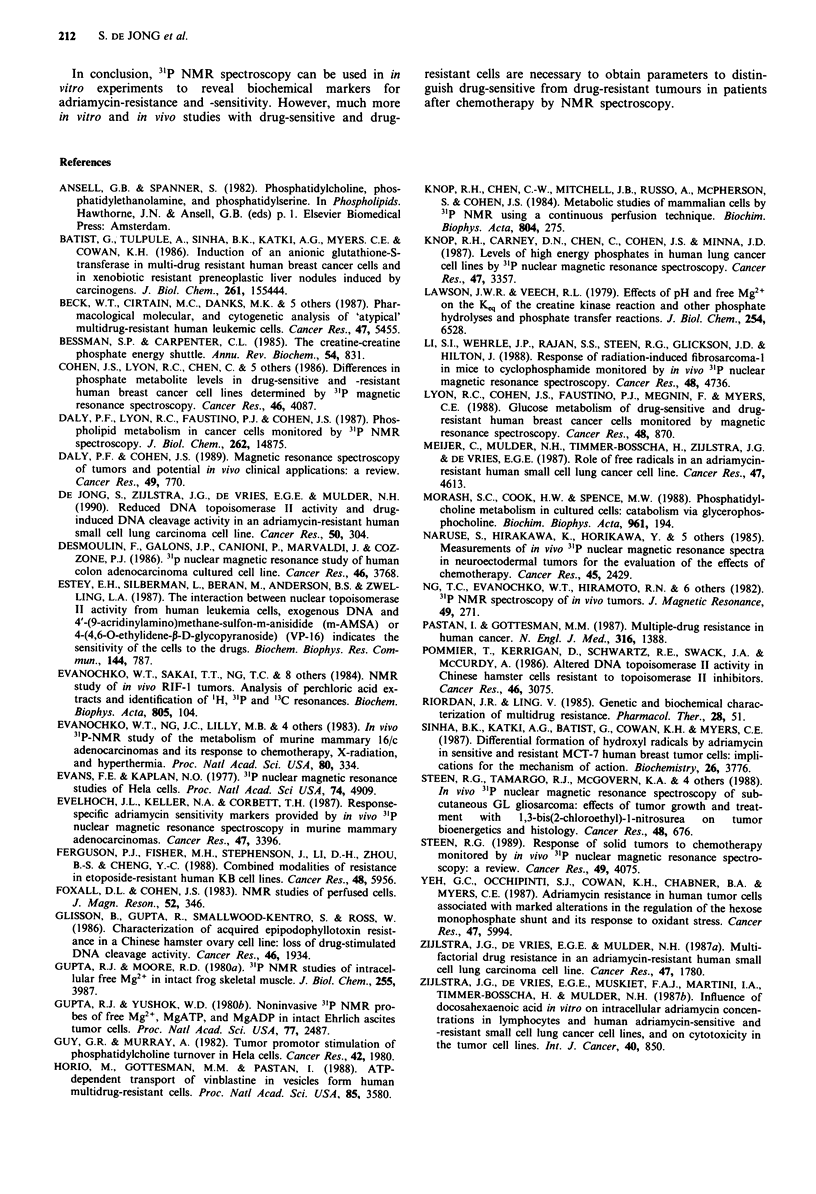# NMR spectroscopy analysis of phosphorus metabolites and the effect of adriamycin on these metabolite levels in an adriamycin-sensitive and -resistant human small cell lung carcinoma cell line.

**DOI:** 10.1038/bjc.1991.50

**Published:** 1991-02

**Authors:** S. de Jong, N. H. Mulder, E. G. de Vries, G. T. Robillard

**Affiliations:** Department of Medical Oncology, State University of Groningen, The Netherlands.

## Abstract

31P nuclear magnetic resonance (NMR) spectra of cells and of cell extract revealed high levels of phosphorylcholine (PC) and phosphocreatine (PCr) in an adriamycin-resistant human small cell lung carcinoma cell line (GLC4/ADR) and the adriamycin-sensitive parental cell line (GLC4). PCr levels in extracts of GLC4/ADR were increased compared to extracts of GLC4. We estimated that 11% of the total intracellular ATP is not bound to Mg2+ in both cell lines. This value corresponded to an intracellular free Mg2+ of 0.30 mM. The effects of different adriamycin concentrations, 0.05, 1 and 30 microM for GLC4 and 1, 30 and 200 microM for GLC4/ADR, on the phosphorus metabolite levels in continuously perfused cells were monitored. Significant differences between GLC4 and GLC4/ADR included: (a) a strong increase in the beta ATP level in the presence of 30 microM adriamycin in GLC4 only, followed by a fast decrease after 5 h of perfusion. (b) a less dramatic increase in the PC level in GLC4/ADR and an unchanged ATP level in the presence of increasing adriamycin concentrations. (c) an increased GPC level in GLC4/ADR in the presence of adriamycin. The changes in PC and GPC levels in the presence of adriamycin suggested that the phospholipid turnover was increased in GLC4/ADR and could be stimulated in the presence of adriamycin. In both cell lines, PCr levels decreased faster than the ATP levels after adriamycin treatment. Thus, biochemical markers for adriamycin resistance can be detected with NMR spectroscopy. However, more studies are necessary to obtain parameters to distinguish drug-sensitive from drug-resistant tumours in patients by NMR spectroscopy.


Br. J. Cancer (1991), 63, 205-212 © Macmillan Press Ltd., 1991

### NMR Spectroscopy analysis of phosphorus metabolites and the effect of adriamycin on these metabolite levels in an adriamycin-sensitive and -resistant human small cell lung carcinoma cell line*

S. de Jong[1], N.H. Mulder[1], E.G.E. de Vries[1] & G.T. Robillard[2]

[1]*Departments of Medical Oncology and* [2]*Physical Chemistry, State University of Groningen, The Netherlands.*

**Summary** $^{31}P$ nuclear magnetic resonance (NMR) spectra of cells and of cell extracts revealed high levels of phosphorylcholine (PC) and phosphocreatine (PCr) in an adriamycin-resistant human small cell lung carcinoma cell line ($GLC_4$/ADR) and the adriamycin-sensitive parental cell line ($GLC_4$). PCr levels in extracts of $GLC_4$/ADR were increased compared to extracts of $GLC_4$. We estimated that 11% of the total intracellular ATP is not bound to $Mg^{2+}$ in both cell lines. This value corresponded to an intracellular free $Mg^{2+}$ of 0.30 mM. The effects of different adriamycin concentrations, 0.05, 1 and 30 μM for $GLC_4$ and 1, 30 and 200 μM for $GLC_4$/ADR, on the phosphorus metabolite levels in continuously perfused cells were monitored. Significant differences between $GLC_4$ and $GLC_4$/ADR included: (a) a strong increase in the βATP level in the presence of 30 μM adriamycin in $GLC_4$ only, followed by a fast decrease after 5 h of perfusion. (b) a less dramatic increase in the PC level in $GLC_4$/ADR and an unchanged ATP level in the presence of increasing adriamycin concentrations. (c) an increased GPC level in $GLC_4$/ADR in the presence of adriamycin. The changes in PC and GPC levels in the presence of adriamycin suggested that the phospholipid turnover was increased in $GLC_4$/ADR and could be stimulated in the presence of adriamycin. In both cell lines, PCr levels decreased faster than the ATP levels after adriamycin treatment. Thus, biochemical markers for adriamycin resistance can be detected with NMR spectroscopy. However, more studies are necessary to obtain parameters to distinguish drug-sensitive from drug-resistant tumours in patients by NMR spectroscopy.

Changes in energy-metabolism may be involved in resistance. Cell lines selected *in vitro* for resistance to adriamycin, Vinca alkaloid or colchicine, exhibit the multidrug resistant (MDR) phenotype. In these resistant cells a $M_r$ 170,000 kD P-glycoprotein is overexpressed (Riordan & Ling, 1985; Pastan & Gottesman, 1987). This P-glycoprotein functions as a energy-dependent efflux pump to different types of antitumour drugs (Riordan & Ling, 1985; Horio *et al.*, 1988). Increased free radical detoxification could also play a role in adriamycin-resistance of these cells (Batist *et al.*, 1986; Sinha *et al.*, 1987). Since both mechanisms are associated with energy-dependent processes, expressing the MDR phenotype may involve changes in energy requirements and energy metabolism. These changes have actually been observed in an adriamycin-resistant human breast cancer cell line (Cohen *et al.*, 1986; Yeh *et al.*, 1987; Lyon *et al.*, 1988). Cell lines resistant to epipodophyllotoxins, ellipticine and m-AMSA have also been established which do not overexpress the P-glycoprotein (Glisson *et al.*, 1986; Estey *et al.*, 1987; Pommier *et al.*, 1986; Beck *et al.*, 1987; Ferguson *et al.*, 1988). Cross resistance to other drugs is still observed and sometimes drug accumulation is decreased. It is unknown whether this so-called atypical MDR (Beck *et al.*, 1987) is accompanied by changes in energy requirements and energy metabolism.

In order to obtain biochemical characteristics for the atypical MDR phenotype we focussed on metabolites of both energy metabolism (PCr, ATP and Pi) and phospholipid metabolism (GPC, PE and PC) in an adriamycin-sensitive small cell lung carcinoma cell line ($GLC_4$) and an adriamycin-resistant subline ($GLC_4$/ADR), which exhibits the atypical MDR phenotype (Zijlstra *et al.*, 1987*a*; De Jong *et al.*, 1990). Phosphorus metabolite levels in living cells can be monitored by $^{31}P$ NMR spectroscopy. So far, studies on the effect of adriamycin exposure on energy and lipid metabolism using $^{31}P$ NMR were only done in *in vivo* models of murine mammary 16/C and murine mammary 17/C adenocarcinomas (Evanochko *et al.*, 1983; Evelhoch *et al.*, 1987). *In vitro* studies on cells using NMR spectroscopy necessitate trapping a dense cell suspension in a small volume. We have used the method described by Cohen *et al.* (1986) in which cells were embedded in agarose gel threads (Foxall & Cohen, 1983; Knop *et al.*, 1984). They have applied their technique using $^{31}P$ and $^{13}C$ NMR spectroscopy to various cell lines (Cohen *et al.*, 1986; Lyon *et al.*, 1988; Daly *et al.*, 1987). In their studies with small cell lung cancers the signal intensities of PCR did not change relative to the ATP signal intensities for over 24 h (Knop *et al.*, 1987).

In the present study, $^{31}P$ and $^{1}H$ NMR spectroscopy was employed to monitor levels of energy and phospholipid metabolism in $GLC_4$ and $GLC_4$/ADR cells. The effect of adriamycin on these levels were monitored in continuously perfused cells using $^{31}P$ NMR spectroscopy. The presence of phosphorus metabolites characteristic for atypical MDR and the presence of response-specific markers of adriamycin-sensitivity and -resistance are discussed.

### Materials and methods

### *Materials*

RPMI 1640 medium was purchased from Gibco (Paisley, Scotland). Dulbecco's Modified Eagles's medium (DME), F12 medium and foetal calf serum (FCS) were obtained from Flow Lab (Irvine, Scotland), low melting agarose from FMC (Rockland, ME) and adriamycin from Farmitalia Carlo Erba (Milano, Italy).

### *Cell lines*

$GLC_4$, a human small cell lung carcinoma cell line, was derived from a pleural effusion in our laboratory and kept in continuous culture in RPMI 1640 medium supplemented with 10% FCS. $GLC_4$/ADR, a subline of the parental line, was made resistant by stepwise increasing concentrations of adriamycin, until the cells were growing at a continuous drug level of 1.18 μM. $GLC_4$/ADR was 44-fold more resistant to adriamycin than $GLC_4$ after a 1 h exposure in the clonogenic assay (Zijlstra *et al.*, 1987*a*). $GLC_4$/ADR exhibited cross-resistance to several other drugs (Zijlstra *et al.*, 1987*a*; De

*This work was supported by the Dutch Cancer Foundation, Koningin Wilhelmina Fonds, grant GUKC 86-01.
Correspondence: N.H. Mulder, Department of Internal Medicine, University Hospital, Oostersingel 59, 9713 EZ Groningen, The Netherlands.
Received 6 July 1990; and in revised form 1 October 1990.

Jong *et al.*, 1990; Meijer *et al.*, 1987), while the P-glycoprotein was not overexpressed in $GLC_4$/ADR (De Jong *et al.*, 1990). Prior to experimental use, $GLC_4$/ADR was cultured without adriamycin for 20 days, at which time the resistance factor was maximal (Meijer *et al.*, 1987). Both cell lines grow partly attached, partly floating and were cultured in RPMI 1640 medium supplemented with 10% FCS at 37°C in a humid atmosphere with 5% $CO_2$.

*Cell perfusion*

Cells ($1-1.5 \times 10^8$) were resuspended in DME/F12 medium (pH 7.4) and 10% FCS. The perfusion system was prepared as described previously (Foxall & Cohen, 1983; Knop *et al.*, 1984) with some modifications. Low-gelling agarose (0.8 ml in 0.9% NaCl) was added at 37°C to 1.6 ml of a cell suspension. Agarose strands were extruded under light pressure through a teflon capillary tube (0.5 mm inside diameter) immersed in an ice/water bath into an Wilmad MRS tube (10 mm inside diameter). The gel threads were perfused by aspiration (1.4 ml $min^{-1}$). A low perfusion rate was used in this study, since the stability of the threads decreased in the presence of adriamycin. Consequently, the number of cells embedded in the threads had to be decreased to prevent partial acidification of the cells. A number of layers of nylon gauzes were used instead of a piece of sponge to restrain the threads, resulting in a higher perfusion capacity. The perfusate from a 200 ml reservoir consisted of DME/F12 and 10% FCS supplemented with penicillin (125 U/$ml^{-1}$) and streptomycin (125 U $ml^{-1}$). Oxygen (95%) and $CO_2$ (5%) were bubbled through the perfusate in the reservoir.

*Cell extracts*

Cell extracts were made from $1-2 \times 10^8$ cells. The extraction procedure was performed at 4°C. Ice-cold perchloric acid (10%) was added to the pellet and the cell mixture was vortexed at the beginning and the end of a 20 min period. The extracts were neutralised with KOH, centrifuged to remove the $KClO_4$ precipitate, freeze-dried and redissolved in $D_2O$ (Evans & Kaplan, 1977).

*$^1H$ and $^{31}P$ NMR spectroscopy*

$^{31}P$ NMR spectra (121.4 MHz) of perfused cells at 37°C were obtained on a Varian VXR-300 spectrometer equipped with a VXR 5000 data station. Spectra were usually obtained from 1500 transients with a spectral window of $\pm 4000$ Hz, 4K data points, a 65° pulse angle, a repetition rate of 2.25 s and a line broadening of 20 Hz. All $^{31}P$ chemical shifts in the spectra were set relative to PCr by setting the PCr signal to 0.00 ppm.

$^1H$ NMR spectra of cell extracts were obtained from 1000 scans at 10°C with 90° pulse angle and a repetition time of 3.4 s under HDO decoupled conditions. $^{31}P$ NMR spectra of cell extracts were obtained from 2000 scans at 10°C with a 55° pulse angle and a repetition time of 40 s under proton decoupled conditions. EDTA and diphenylphosphate were added to a final concentration of 10 mM and 0.15 mM, respectively.

Since the relative separations between the $\beta$ and $\alpha$, and the $\beta$ and $\gamma$ peaks of ATP are proportional to the amount of ATP bound to $Mg^{2+}$, the fraction of total ATP that is not complexed to $Mg^{2+}$ (O) can be calculated (Gupta & Moore, 1980*a*). The free $Mg^{2+}$ concentration can then be calculated using the dissociation constant of MgATP (ATP complexed to magnesium) ($K_d = 38\ \mu M$) and the formula $[Mg^{2+}] = K_d (O^{-1} - 1)$ (Gupta & Moore, 1980*a*).

Spectra of perfused cells were obtained 3 h after the perfusion was started, when no major changes in the spectra occurred. Two spectra were collected to estimate peak areas and peak heights at t = 0. Peak intensities of the different metabolites in spectra of cell extracts and in perfused cells were estimated by peak areas determined from computer simulated spectra using the deconvolution routine in the VXR-5000 software. Changes in the levels of the phosphorus metabolites in the presence or absence of adriamycin were estimated from resolution-enhanced spectra by comparing peak heights of the particular metabolite at different times. Peak heights of a given metabolite were expressed as a percent of the averaged peak height of this metabolite in the two spectra at t = 0. At lower fields an underlying 'hump' was absent in the spectra, which allowed the reliable and reproducible measurement of peak intensities. Using peak areas from resolution-enhanced spectra to estimate changes in metabolites did not give significantly different results.

*Statistics*

All results were expressed as means ± s.d. Statistical significance was determined by use of the Student's *t*-test.

### Results

*$^{31}P$ and $^1H$ NMR spectra of cell extracts*

Cell extracts were made from adriamycin-sensitive ($GLC_4$) and adriamycin-resistant ($GLC_4$/ADR) cells. Assignments were made on basis of data in the literature (Daly *et al.*, 1987; Evans & Kaplan, 1977; Evanochko *et al.*, 1984) and by adding standard compounds. Extracts of both cell lines showed high levels of PC (1 in Figure 1a and b). Additional unidentified resonances could be seen, possibly AMP and PE, on the low field side of the PC peaks. Low levels of probably GPC (4) and GPE (3) could be detected in the extracts. High levels of PCr (5) were detected in both cell lines with highest PCr levels in the $GLC_4$/ADR extracts (Table I). Expanding the spectra revealed another triphosphate near the $\beta$ resonances of ATP (11) that accounted for $25 \pm 4\%$ (s.d., $n = 3$) of the total peak area in $GLC_4$/ADR extracts and for $19 \pm 4\%$ in $GLC_4$ extracts. This triphosphate could be UTP, GTP or CTP (Evans & Kaplan, 1977; Evanochko *et al.*, 1984). High resolution $^1H$ NMR spectra were obtained from extracts of both cell lines in $D_2O$. Figure 2 shows the results for $GLC_4$. The identification of the peaks was made using previous assignments in tumour cell extracts and by adding standard compounds (Evanochko *et al.*, 1984). The most intense resonances originated from choline, PC, PCr and creatine; furthermore, lactate, acetate and amino acids (alanine, proline, glutamic acid and glutamine) were found. In the low field region of the $^1H$ NMR spectra resonances from adenosine derivatives and some uracil-, guanine- and cytosine-containing compounds predominated. No major differences between spectra of $GLC_4$ and $GLC_4$/ADR were found.

*$^{31}P$ NMR spectra of perfused cells*

Spectra of perfused $GLC_4$ and $GLC_4$/ADR cells at 37°C were recorded at a 2.25 s repetition rate and a 55° pulse angle to ensure almost complete spin relaxation of the metabolites (Figure 3). To estimate the relative levels of phosphorus metabolites in $GLC_4$ and $GLC_4$/ADR we determined peak areas of the various metabolites (Table II) in several spectra at t = 0 by computer simulation of the spectra using a deconvolution routine. Partially overlapping peaks could be separated using this program. The peak areas were expressed to that of the $\beta$ATP (8) resonance. Peak areas of Pi (2) were not used, since Pi was also present in the medium we used. Peak area of $\gamma$ATP (5) was increased in $GLC_4$/ADR compared to $GLC_4$. Since the peak areas are expressed relative to the $\beta$ATP peak area, the increased area of the $\gamma$ATP resonance in $GLC_4$/ADR must be due to some component other than ATP. Comparing the extracts we concluded that ADP was this component.

*Free ADP and $Mg^{2+}$ in intact cells*

$Mg^{2+}$ modifies the equilibrium constant for the reactions

**Figure 1** $^{31}P$ NMR spectra (121.45 MHz) of the perchloric extracts of $GLC_4$ **a**, and $GLC_4/ADR$ **b**. The pH (meter reading) was 7.8. Peak assignments are: 1, PC; 2, Pi; 3, GPE; 4, GPC; 5, PCr; 6, γATP; 7, βADP; 8, αATP; 9, αATP; 10, NAD; 11, βATP.

**Table I** Phosphorus metabolite levels in extracts of $GLC_4$ and $GLC_4/ADR$ ($n = 3$)

| | *GLC₄* | *GLC₄/ADR* |
|---|---|---|
| PC | 1.83 ± 0.44[a] | 1.41 ± 0.11 |
| GPC | 0.10 ± 0.05 | 0.13 ± 0.04 |
| PCr | 0.71 ± 0.18 | 1.11 ± 0.16[d] |
| γATP[b] | 1.09 ± 0.14 | 1.20 ± 0.07 |
| αATP[b] | 1.56 ± 0.11 | 1.66 ± 0.12 |
| βATP[c] | 1.00 | 1.00 |

[a]Peak areas were obtained from spectra using a deconvolution routine (see Materials and methods) and were expressed relatively to the peak area of βATP (± s.d.), [b]ADP, NAD and some other triphosphates might be present, [c]Some other triphosphates might be present, [d]$P < 0.025$, $GLC_4$ *vs* $GLC_4/ADR$.

**Figure 2** $^{1}H$ NMR spectrum (300 MHz) of the perchloric extract of $GLC_4/ADR$. The pH was 7.4. The amplitude of the peaks in the high field region (5.5–9.5 ppm) is eight times that of the peaks in the low field region (0–4 ppm) A, adenine; G, guanine; C, cytosine; U, uracyl; PCr, phosphocreatine; Cr, creatine; PC + C, phosphorylcholine and choline; Pro, proline; Glu, glutamic acid; Gln, glutamine; Ala, alanine; Lac, lactate.

**Figure 3** $^{31}P$ NMR spectra (121.45 MHz) of perfused $GLC_4$ **a**, and $GLC_4/ADR$ **b**, cells at 37°C. Peak assignments are: 1, PC; 2, Pi; 3, GPC; 4, PCr; 5, γATP; 6, αATP; 7, NAD; 8, βATP.

**Table II** Phosphorus metabolite levels in perfused $GLC_4$ and $GLC_4/ADR$ cells ($n = 6$)

| | *GLC₄* | *GLC₄/ADR* |
|---|---|---|
| PC | 1.96 ± 0.35 | 1.94 ± 0.29 |
| GPC | 0.08 ± 0.09 | 0.09 ± 0.13 |
| PCr | 0.64 ± 0.18 | 0.54 ± 0.14 |
| γATP[b] | 1.03 ± 0.15 | 1.27 ± 0.21[d] |
| αATP[b] | 1.50 ± 0.26 | 1.76 ± 0.34 |
| βATP[c] | 1.00 | 1.00 |

[a]Peak areas were obtained from spectra at $t = 0$ using a deconvolution routine (see Materials and methods) and were expressed relatively to the peak area of βATP (± s.d.), [b]ADP, NAD and some other triphosphates might be present, [c]Some other triphosphates might be present, [d]$P < 0.05$, $GLC_4$ *vs* $GLC_4/ADR$.

**Figure 4** Effect of different adriamycin concentrations on PCr levels in perfused $GLC_4$ **a**, and $GLC_4$/ADR **b**, cells at 37°C. After the $^{31}P$ NMR spectra at t = 0 were obtained, adriamycin was added to the perfusate. Levels were obtained from peak heights of the metabolite in the $^{31}P$ NMR spectra, as described in Materials and methods, **a**, control (——O——), 0.05 μM (--●--), 1 μM (—■—) and 30 μM (·-◆-·) adriamycin, **b**, control (——O——), 1 μM (—■—), 30 μM (·-◆-·) and 200 μM (··-*-··) adriamycin. Points, mean of three experiments, bars, s.d.

catalysed by creatine kinase and adenylate kinase that are important for energy metabolism (Lawson & Veech, 1979). The spectra of perfused cells we had used to quantify relative levels of metabolites were also used to estimate the fraction of total ATP not bound to $Mg^{2+}$. This fraction was 0.11 ± 0.01 in $GLC_4$/ADR and 0.11 ± 0.02 in $GLC_4$ cells. The free intracellular $Mg^{2+}$ concentrations were calculated from these fractions as described in Materials and methods and were 0.32 ± 0.03 mM and 0.30 ± 0.06 mM in $GLC_4$/ADR and $GLC_4$. The total ADP concentration (MgADP and free ADP) can be calculated from ATP, PCr and Cr concentrations in extracts, the intracellular pH and the assumed equilibrium constant $K_{ck}$ of the creatine kinase reaction at this $Mg^{2+}$ concentration according to Lawson et al. (1979). The ATP concentration in extracts of both cell lines was 6 nmol/$10^6$ cells (De Jong et al., manuscript in preparation). The Cr/PCr ratio was calculated from $^1H$ NMR spectra of extracts. For $GLC_4$ and $GLC_4$/ADR these ratios were 1.33 ± 0.12 (s.d., $n$ = 3) and 1.39 ± 0.37, respectively. Assuming that the intracellular and extracellular pH are equal (pH 7.3), the calculated total ADP concentration was ≈0.15 nmol/$10^6$ cells for $GLC_4$ and $GLC_4$/ADR.

### *Effect of adriamycin on energy metabolite levels of perfused $GLC_4$ and $GLC_4$/ADR cells*

Perfused cells were continuously exposed to 0.05 μM, 1 μM and 30 μM adriamycin ($GLC_4$) and to 1 μM, 30 μM and 200 μM adriamycin ($GLC_4$/ADR) while the time course of the phosphorus metabolite levels was followed. Each $^{31}P$ NMR spectrum was obtained by accumulating 1500 scans which took 1 h. Only significant changes are indicated.

PCr levels in the control (untreated $GLC_4$ cells) increased to 130% of the initial value (t = 2–12 h, $P < 0.05$ vs t = 0) (Figure 4a). In the presence of 30 μM adriamycin, PCr levels decreased rapidly after 4 h (t = 5–15 h, $P < 0.01$ vs control) and were almost undetectable at 15 h. In untreated $GLC_4$/ADR cells PCr levels did not increase significantly of the initial value, while with a high concentration of adriamycin (200 μM) PCr levels decreased after 4 h (t = 5–15 h, $P < 0.005$ vs control) and became undetectable after 13 h (Figure 4b). The standard deviations of PCr levels were rather large in adriamycin treated cells of both cell lines because the peak intensity was low and therefore more susceptible to noise.

Since the γATP and αATP resonances might contain some contributions from ADP, changes in height of the βATP resonance were used to determine the influence of adriamycin on ATP. ATP levels in untreated $GLC_4$ cells increased to 140% of the initial value (t = 4–15 h, $P < 0.05$ vs t = 0) (Figure 5a). In the presence of 30 μM adriamycin an increase to 175% of the initial ATP level was seen within 5 to 6 h (t = 2–7 h, $P < 0.025$ vs control), which subsequently decreased to 20% at 15 h (Figure 5a and 6a). In untreated $GLC_4$/ADR cells ATP levels increased to 125% of the initial value (t = 3–15 h, $P < 0.05$ vs t = 0) (Figure 5b). With 200 μM adriamycin ATP dropped to undetectable levels at 15 h (t = 6–15 h, $P < 0.01$ vs control). Changes in γATP and αATP intensities in untreated and adriamycin treated $GLC_4$

**Figure 5** Effect of different adriamycin concentrations on βATP levels in perfused $GLC_4$ **a**, and $GLC_4$/ADR **b**, cells at 37°C. After the $^{31}P$ NMR spectra at t = 0 h were obtained, adriamycin was added to the perfusate. Levels were obtained from peak heights of the metabolite in the $^{31}P$ NMR spectra, as described in Materials and methods. **a**, control (——O——), 0.5 μM (--●--), 1 μM (——■——) and 30 μM) (·-◆-·) adriamycin, **b**, control (——O——), 1 μM (——■——), 30 μM (·-◆-·) and 200 μM (··-*-··) adriamycin. Points, mean of three experiments, bars, s.d.

**Figure 6** $^{31}P$ NMR spectra (121.45 MHz) of perfused $GLC_4$ **a**, and $GLC_4$/ADR, **b**, cells at different intervals in the presence of 30 μM adriamycin. Adriamycin was added to the perfusate directly after the spectra at t = 0 h were obtained. Peak assignments are as described in legend of Figure 3.

and $GLC_4$/ADR cells were almost identical to changes in βATP intensity (results not shown). Neither the percentage of unbound ATP nor the intracellular $Mg^{2+}$ concentration changed in the presence of various concentrations of adriamycin.

The energy status of a cell could be described by the PCr/βATP ratio. We averaged the results from 1 to 5 h, 6 to 10 h and 11 to 15 h for the different adriamycin concentrations used (Figure 7). From this figure it could be concluded that the PCr level decreased faster than the βATP level in both cell lines in response to high adriamycin concentrations, 30 μM for $GLC_4$ (t = 5–15 h, $P < 0.01$ *vs* control) and 200 μM for $GLC_4$/ADR (t = 10–15 h, $P < 0.025$ *vs* control). With 0.05 μM adriamycin the PCr level decreased faster than the βATP level in $GLC_4$ cells (t = 10–15 h, $P < 0.05$ *vs* control).

*Effect of adriamycin on phospholipid metabolite levels of perfused $GLC_4$ and $GLC_4$/ADR cells*

PC levels in untreated $GLC_4$ cells increased to 170% of the initial value (t = 2–15 h, $P < 0.05$ *vs* control) (Figure 8a). In the presence of 30 μM adriamycin the level slowly dropped to 40% of the initial value (t = 5–15 h, $P < 0.005$ *vs* control) (Figures 6a and 8a). In untreated $GLC_4$/ADR cells PC levels increased to 150% (t = 3–15 h, $P < 0.05$ *vs* t =) (Figure 8b). In the presence of increasing concentrations of adriamycin PC levels increased less compared to levels in untreated cells (30 μM adriamycin, t = 7–15 h, $P < 0.01$ *vs* control) and even decreased in the presence of 200 μM adriamycin (t = 3–15 h, $P < 0.025$ *vs* control). The low intensity made the level of GPC difficult to estimate; consequently the results were averaged. Levels of GPC were expressed as percentage of the initial peak height of βATP at 0 h, since GPC levels were sometimes undetectable in the 0 h spectra. GPC peaks were almost undetectable in $GLC_4$ and did not change in the presence of adriamycin (Figures 3a and 6a). GPC levels in $GLC_4$/ADR increased from 37 ± 9% (s.d.) in untreated cells to 63 ± 17% ($P < 0.05$ *vs* control) in 1 μM adriamycin treated cells and to 76 ± 29% ($P < 0.05$ *vs* control) in 30 μM treated cells (Figures 3b and 6b). In the presence of 200 μM adriamycin GPC levels increased to 62 ± 6% ($P < 0.005$ *vs* control) and after 8 h decreased to control values.

*Extracts of adriamycin treated cells*

Extracts were made of cells treated with adriamycin to examine the possibility that changes in components as estimated in intact cells spectra were actually due to the appearance of new components. After treatment of $GLC_4$ cells and $GLC_4$/ADR cells with 1 and 30 μM adriamycin for 5 h similar results were obtained in extracts as in perfused cells, while no new components were detected in the spectra (results not shown). Extracts were made from control $GLC_4$ cells and from cells continuously incubated with 0.05 μM and 1 μM adriamycin for 15 h. Two unassigned components, probably PE and AMP, were clearly visible to the low field of PC (1) in control $GLC_4$ cell spectra (Figure 9a), that disapppeared after treatment with 1 μM adriamycin (results not shown). No new components were detected in extract spectra of control $GLC_4$/ADR cells (Figure 9b) and extract spectra from cells after treatment with 1 and 30 μM adriamycin for 15 h. $^1H$ NMR spectra of these extracts showed no changes at all in the presence of adriamycin (results not shown).

**Figure 7** Effect of different adriamycin concentrations on the PCr/βATP ratio in perfused $GLC_4$ **a**, and $GLC_4$/ADR **b**, cells at 37°C. After the $^{31}P$ NMR spectra at t = 0 h were obtained, adriamycin was added to the perfusate. Ratios were obtained from peak heights of the metabolites in the $^{31}P$ NMR spectra, as described in Materials and methods and Results.

### Discussion

$^{31}P$ NMR spectra from perfused cells and extracts showed the same resonances. Several cellular compartments have been described such as mitochondria that might influence the peak intensity of βATP (Gupta & Yushok, 1980*b*). However, phosphorus metabolite content in cellular extracts as determined by $^{31}P$ NMR and biochemical analysis are in agreement (Desmoulin *et al.*, 1986). Furthermore, since spectral resolution was enhanced and complete relaxation of the phosphorus resonances spectra was obtained in our extracts, the significance of differences observed in peak areas relative to the βATP peak area is better indicated by comparing cell extracts. The relative PCr concentration was higher in extracts of $GLC_4$/ADR compared to $GLC_4$, however the ratio PCr/Cr was not changed. The percentage of unbound ATP and the intracellular $Mg^{2+}$ concentration were similar in intact $GLC_4$ and $GLC_4$/ADR cells. These results indicate that the equilibrium constant for the creatine kinase reaction and the equilibrium of this reaction are equal for both cell lines. The calculated intracellular $Mg^{2+}$ concentration of 0.3 mM was comparable with the intracellular $Mg^{2+}$ concentration of 0.4 mM in Ehrlich ascites tumour cells (Gupta & Yushok, 1980*b*). The relatively higher PCr level in $GLC_4$/ADR could increase the capacity of these cells to maintain the ATP pool. In a previous report, high levels of PCr and low levels of disphosphodiester were observed in variant SCLC cell lines compared to classic SCLC cell lines (Knop *et al.*, 1987). Thus, the presence of high levels of PCr and the absence of diphosphodiester support our earlier characterisation of these two cell lines as variant SCLC cell lines (Zijlstra *et al.*, 1987*a*).

PC was observed in cell extracts as well as intact cells (Figures 1–3). The PE resonance was not present in the spectra, since these cells were grown in media without ethanolamine. However, these cells can still produce phosphatidylethanolamine by decarboxylation of phosphatidylserine (Daly *et al.*, 1987; Ansell & Spanner, 1982). Phospholipid analysis of our cell lines indeed revealed the presence of phosphatidylethanolamine (Zijlstra *et al.*, 1987*b*). The presence of PC and PE in tumour cells might be of diagnostic value, since *in vivo* human tumours showed elevated levels of PC and PE compared to the tissue of origin (Daly & Cohen, 1989).

In adriamycin-resistant MCF-7 breast cancer cells, PCr levels were increased, while PC, GPC, GPE and diphosphodiester levels were decreased compared to the ATP level (in the original report the PC and PE peaks were assigned to sugar phosphates) (Cohen *et al.*, 1986). NMR studies of other cell lines indicated that differences observed in metabolite levels did not correlate specifically with drug-resistance (Evelhoch *et al.*, 1987). Decreased GPC, PC and PE levels were also observed in *in vivo* adriamycin-resistant 17/A adenocarcinomas, but as the untreated tumours progressed, the differences between the adriamycin-sensitive and -resistant tumours disappeared (Evelhoch *et al.*, 1987). Therefore, it is uncertain whether differences in phosphorus metabolite levels are related to the MDR or the atypical MDR phenotype.

Both $GLC_4$ and $GLC_4$/ADR are anchorage-independent cell lines. Since an increase in PC could be related to an

**Figure 8** Effect of different adriamycin concentrations on PC levels in perfused $GLC_4$ **a**, and $GLC_4$/ADR **b**, cells at 37°C. After the $^{31}P$ NMR spectra at t = 0 h were obtained, adriamycin was added to the perfusate. Levels were obtained from peak heights of the metabolite in the $^{31}P$ NMR spectra as described in Materials and methods, **a**, control (——○——), 0.5 μM (– –●– –), 1 μM (——■——) and 30 μM (· –◆– ·) adriamycin, **b**, control (——○——), 1 μM (——■——), 30 μM (· –◆– ·) and 200 μM (· · –*– · ·) adriamycin. Points, mean of three experiments, bars, s.d.

**Figure 9** $^{31}P$ NMR spectra (121.45 MHz) of the perchloric extracts of $GLC_4$, **a**, and $GLC_4$/ADR **b**, after 15 h. The pH was 7.8. Peak assignments are as described in the legend of Figure 1.

increased cell growth (Daly *et al.*, 1987), the significant increase in PC and ATP in the control experiments with continuous perfused cells was probably due to cell growth. Furthermore, the perfusion experiments showed that several phosphorus metabolites were response-specific biochemical markers of adriamycin sensitivity and resistance. When $GLC_4$ cells were treated with 30 $\mu$M adriamycin ATP levels increased faster than levels in untreated cells. This increase could either be due to a decreased energy consumption or an increased energy production. Even treatment with 200 $\mu$M adriamycin did not result in a strong increase of the ATP level in $GLC_4$/ADR, although the level dropped to undetectable during the experiment in a way similar to ATP levels in $GLC_4$ cells treated with 30 $\mu$M adriamycin. In both cell lines the PCr/ATP ratio decreased in response to high adriamycin concentrations. PCr was probably used to maintain the ATP pool at a stable level via creatine kinase as described in muscle (Bessman & Carpenter, 1985).

An interesting finding was the effect of adriamycin treatment on the PC and GPC level only in $GLC_4$/ADR cells. We could not confirm the increase in GPC levels in extracts. There are two possibilities. First, by using the continuous perfusion system $GLC_4$/ADR cells were physically stressed resulting in an increase in phospholipid turnover that was stimulated by adriamycin treatment. Secondly, the peak we saw, was not due to GPC, but to a phospholipid component also resulting from an increased phospholipid turnover. In phospholipid synthesis, choline is converted to PC and further converted to phosphatidylcholine (Ansell & Spanner, 1982). This phospholipid is degradaded to GPC and then to choline by glycerophosphocholine phosphodiesterase (EC 3.1.4.2) (Ansell & Spanner, 1982; Morash *et al.*, 1988). The increased phospholipid turnover might be related to the reduced adriamycin accumulation in $GLC_4$/ADR cells. The reduced drug accumulation was not due to the increased activity of the P-glycoprotein (Zijlstra *et al.*, 1987*a*; De Jong *et al.*, 1990).

In human and rat neuroectodermal tumours ATP levels decreased strongly within 6 to 12 h after cyclophosphamide, vincristine and methotrexate treatment, while PCr levels remained undetectable (Naruse *et al.*, 1985). In MOPC 104E myeloma PCr/ATP ratio increased within 1 day after treatment with a curative dose of cyclophosphamide or 1,3-bis(2-chloroethyl)-1-nitrosourea, while PCr and ATP levels strongly reduced within 4 days (Ng *et al.*, 1982). In this study it was concluded, that the changed PCr/ATP ratio must partially reflect the effect of the chemotherapy on energy metabolism within the tumour cells. ATP/Pi and PCr/Pi ratios in adriamycin-sensitive mammary 17/A adenocarcinoma (Evelhoch *et al.*, 1987), in the RIF-1 fibrosarcoma (Li *et al.*, 1988) and in GL gliosarcoma (Steen *et al.*, 1988) were increased after adriamycin, cyclophosphamide and 1,3-bis(2-chloroethyl)-1-nitrosourea treatment, respectively. The increase of these ratios after treatment was explained by reenergization of the tumour, while untreated control tumours in these studies showed declining ATP and PCr levels (Evelhoch *et al.*, 1987; Li *et al.*, 1988; Steen *et al.*, 1988). Untreated neuroectodermal tumours were still in an active stage which may explain the fast reduction in ATP levels 3 h after treatment with cyclophosphamide (300 mg $kg^{-1}$) (Naruse *et al.*, 1985), while an opposite effect of cyclophosphamide (300 mg $kg^{-1}$) was seen in RIF-1 fibrosarcoma (Li *et al.*, 1988). Untreated MOPC 104E myelomas were in a moderate active stage which may explain the slow decrease in ATP levels in 4 days (Ng *et al.*, 1982).

In our *in vitro* experiments the continuous perfused cells were supplied with sufficient nutrients. Therefore, these results showed without any interference from reenergization that adriamycin treatment had an effect on the energy metabolism in the adriamycin-sensitive $GLC_4$ tumour cells which resulted in an increase of cellular ATP. The strong decrease in ATP and PCr levels after treatment were comparable with the effects of chemotherapy in *in vivo* tumours that were in a metabolic active stage (Ng *et al.*, 1982; Naruse *et al.*, 1985). The same adriamycin concentration had no effect on ATP and PCr levels in the adriamycin-resistant $GLC_4$/ADR tumour cells compared to untreated $GLC_4$/ADR cells. No differences in phosphorus metabolites were observed in adriamycin treated and untreated adriamycin-resistant mammary 17/A adenocarcinoma, while adriamycin had a large effect on the adriamycin-sensitive tumour (Evelhoch *et al.*, 1987). To distinguish drug-sensitive from drug-resistant tumours in patients, it will be necessary to compare changes in phosphorus metabolite levels in tumours after treatment with an estimation of the changes in nucleoside triphosphates and PCr levels in this tumour that would occur without chemotherapeutic treatment, since the metabolic stage of a tumour probably determines the changes in phosphorus metabolites after chemotherapy. To obtain a reliable estimation, tissue heterogenity, tumour size, type of tumour, the glycolytic rate of the tumour, tumour hypoxia and the degree of vascularisation of a tumour have to be determined. Till now only a few often preliminary data are available on human tumour bioenergetics and responses to chemotherapy observed by NMR (Steen, 1989). The continuous perfusion system can be used to study the relation between hypoxia and/or glucose deprivation and chemotherapeutic effectivity in *in vitro* experiments.

In conclusion, $^{31}P$ NMR spectroscopy can be used in *in vitro* experiments to reveal biochemical markers for adriamycin-resistance and -sensitivity. However, much more *in vitro* and *in vivo* studies with drug-sensitive and drug-resistant cells are necessary to obtain parameters to distinguish drug-sensitive from drug-resistant tumours in patients after chemotherapy by NMR spectroscopy.

### References

ANSELL, G.B. & SPANNER, S. (1982). Phosphatidylcholine, phosphatidylethanolamine, and phosphatidylserine. In *Phospholipids*. Hawthorne, J.N. & Ansell, G.B. (eds) p. 1. Elsevier Biomedical Press: Amsterdam.

BATIST, G., TULPULE, A., SINHA, B.K., KATKI, A.G., MYERS. C.E. & COWAN, K.H. (1986). Induction of an anionic glutathione-S-transferase in multi-drug resistant human breast cancer cells and in xenobiotic resistant preneoplastic liver nodules induced by carcinogens. *J. Biol. Chem.*, **261**, 155444.

BECK, W.T., CIRTAIN, M.C., DANKS, M.K. & 5 others (1987). Pharmacological molecular, and cytogenetic analysis of 'atypical' multidrug-resistant human leukemic cells. *Cancer Res.*, **47**, 5455.

BESSMAN, S.P. & CARPENTER, C.L. (1985). The creatine-creatine phosphate energy shuttle. *Annu. Rev. Biochem.*, **54**, 831.

COHEN, J.S., LYON, R.C., CHEN, C. & 5 others (1986). Differences in phosphate metabolite levels in drug-sensitive and -resistant human breast cancer cell lines determined by $^{31}P$ magnetic resonance spectroscopy. *Cancer Res.*, **46**, 4087.

DALY, P.F., LYON, R.C., FAUSTINO, P.J. & COHEN, J.S. (1987). Phospholipid metabolism in cancer cells monitored by $^{31}P$ NMR spectroscopy. *J. Biol. Chem.*, **262**, 14875.

DALY, P.F. & COHEN, J.S. (1989). Magnetic resonance spectroscopy of tumors and potential *in vivo* clinical applications: a review. *Cancer Res.*, **49**, 770.

DE JONG, S., ZIJLSTRA, J.G., DE VRIES, E.G.E. & MULDER, N.H. (1990). Reduced DNA topoisomerase II activity and drug-induced DNA cleavage activity in an adriamycin-resistant human small cell lung carcinoma cell line. *Cancer Res.*, **50**, 304.

DESMOULIN, F., GALONS, J.P., CANIONI, P., MARVALDI, J. & COZZONE, P.J. (1986). $^{31}p$ nuclear magnetic resonance study of human colon adenocarcinoma cultured cell line. *Cancer Res.*, **46**, 3768.

ESTEY, E.H., SILBERMAN, L., BERAN, M., ANDERSON, B.S. & ZWELLING, L.A. (1987). The interaction between nuclear topoisomerase II activity from human leukemia cells, exogenous DNA and 4'-(9-acridinylamino)methane-sulfon-m-anisidide (m-AMSA) or 4-(4,6-O-ethylidene-β-D-glycopyranoside) (VP-16) indicates the sensitivity of the cells to the drugs. *Biochem. Biophys. Res. Commun.*, **144**, 787.

EVANOCHKO, W.T., SAKAI, T.T., NG, T.C. & 8 others (1984). NMR study of *in vivo* RIF-1 tumors. Analysis of perchloric acid extracts and identification of $^{1}H$, $^{31}P$ and $^{13}C$ resonances. *Biochem. Biophys. Acta*, **805**, 104.

EVANOCHKO, W.T., NG, J.C., LILLY, M.B. & 4 others (1983). *In vivo* $^{31}P$-NMR study of the metabolism of murine mammary 16/c adenocarcinomas and its response to chemotherapy, X-radiation, and hyperthermia. *Proc. Natl Acad. Sci. USA*, **80**, 334.

EVANS, F.E. & KAPLAN, N.O. (1977). $^{31}P$ nuclear magnetic resonance studies of Hela cells. *Proc. Natl Acad Sci USA*, **74**, 4909.

EVELHOCH, J.L., KELLER, N.A. & CORBETT, T.H. (1987). Response-specific adriamycin sensitivity markers provided by *in vivo* $^{31}P$ nuclear magnetic resonance spectroscopy in murine mammary adenocarcinomas. *Cancer Res.*, **47**, 3396.

FERGUSON, P.J., FISHER, M.H., STEPHENSON, J., LI, D.-H., ZHOU, B.-S. & CHENG, Y.-C. (1988). Combined modalities of resistance in etoposide-resistant human KB cell lines. *Cancer Res.*, **48**, 5956.

FOXALL, D.L. & COHEN, J.S. (1983). NMR studies of perfused cells. *J. Magn. Reson.*, **52**, 346.

GLISSON, B., GUPTA, R., SMALLWOOD-KENTRO, S. & ROSS, W. (1986). Characterization of acquired epipodophyllotoxin resistance in a Chinese hamster ovary cell line: loss of drug-stimulated DNA cleavage activity. *Cancer Res.*, **46**, 1934.

GUPTA, R.J. & MOORE, R.D. (1980*a*). $^{31}P$ NMR studies of intracellular free $Mg^{2+}$ in intact frog skeletal muscle. *J. Biol. Chem.*, **255**, 3987.

GUPTA, R.J. & YUSHOK, W.D. (1980*b*). Noninvasive $^{31}P$ NMR probes of free $Mg^{2+}$, MgATP, and MgADP in intact Ehrlich ascites tumor cells. *Proc. Natl Acad. Sci. USA*, **77**, 2487.

GUY, G.R. & MURRAY, A. (1982). Tumor promotor stimulation of phosphatidylcholine turnover in Hela cells. *Cancer Res.*, **42**, 1980.

HORIO, M., GOTTESMAN, M.M. & PASTAN, I. (1988). ATP-dependent transport of vinblastine in vesicles form human multidrug-resistant cells. *Proc. Natl Acad. Sci. USA*, **85**, 3580.

KNOP, R.H., CHEN, C.-W., MITCHELL, J.B., RUSSO, A., MCPHERSON, S. & COHEN, J.S. (1984). Metabolic studies of mammalian cells by $^{31}P$ NMR using a continuous perfusion technique. *Biochim. Biophys. Acta*, **804**, 275.

KNOP, R.H., CARNEY, D.N., CHEN, C., COHEN, J.S. & MINNA, J.D. (1987). Levels of high energy phosphates in human lung cancer cell lines by $^{31}P$ nuclear magnetic resonance spectroscopy. *Cancer Res.*, **47**, 3357.

LAWSON, J.W.R. & VEECH, R.L. (1979). Effects of pH and free $Mg^{2+}$ on the $K_{eq}$ of the creatine kinase reaction and other phosphate hydrolyses and phosphate transfer reactions. *J. Biol. Chem.*, **254**, 6528.

LI, S.I., WEHRLE, J.P., RAJAN, S.S., STEEN, R.G., GLICKSON, J.D. & HILTON, J. (1988). Response of radiation-induced fibrosarcoma-1 in mice to cyclophosphamide monitored by *in vivo* $^{31}P$ nuclear magnetic resonance spectroscopy. *Cancer Res.*, **48**, 4736.

LYON, R.C., COHEN, J.S., FAUSTINO, P.J., MEGNIN, F. & MYERS, C.E. (1988). Glucose metabolism of drug-sensitive and drug-resistant human breast cancer cells monitored by magnetic resonance spectroscopy. *Cancer Res.*, **48**, 870.

MEIJER, C., MULDER, N.H., TIMMER-BOSSCHA, H., ZIJLSTRA, J.G. & DE VRIES, E.G.E. (1987). Role of free radicals in an adriamycin-resistant human small cell lung cancer cell line. *Cancer Res.*, **47**, 4613.

MORASH, S.C., COOK, H.W. & SPENCE, M.W. (1988). Phosphatidylcholine metabolism in cultured cells: catabolism via glycerophosphocholine. *Biochim. Biophys. Acta*, **961**, 194.

NARUSE, S., HIRAKAWA, K., HORIKAWA, Y. & 5 others (1985). Measurements of *in vivo* $^{31}P$ nuclear magnetic resonance spectra in neuroectodermal tumors for the evaluation of the effects of chemotherapy. *Cancer Res.*, **45**, 2429.

NG, T.C., EVANOCHKO, W.T., HIRAMOTO, R.N. & 6 others (1982). $^{31}P$ NMR spectroscopy of *in vivo* tumors. *J. Magnetic Resonance*, **49**, 271.

PASTAN, I. & GOTTESMAN, M.M. (1987). Multiple-drug resistance in human cancer. *N. Engl. J. Med.*, **316**, 1388.

POMMIER, T., KERRIGAN, D., SCHWARTZ, R.E., SWACK, J.A. & MCCURDY, A. (1986). Altered DNA topoisomerase II activity in Chinese hamster cells resistant to topoisomerase II inhibitors. *Cancer Res.*, **46**, 3075.

RIORDAN, J.R. & LING. V. (1985). Genetic and biochemical characterization of multidrug resistance. *Pharmacol. Ther.*, **28**, 51.

SINHA, B.K., KATKI, A.G., BATIST, G., COWAN, K.H. & MYERS, C.E. (1987). Differential formation of hydroxyl radicals by adriamycin in sensitive and resistant MCT-7 human breast tumor cells: implications for the mechanism of action. *Biochemistry*, **26**, 3776.

STEEN, R.G., TAMARGO, R.J., MCGOVERN, K.A. & 4 others (1988). *In vivo* $^{31}P$ nuclear magnetic resonance spectroscopy of subcutaneous GL gliosarcoma: effects of tumor growth and treatment with 1,3-bis(2-chloroethyl)-1-nitrosurea on tumor bioenergetics and histology. *Cancer Res.*, **48**, 676.

STEEN, R.G. (1989). Response of solid tumors to chemotherapy monitored by *in vivo* $^{31}P$ nuclear magnetic resonance spectroscopy: a review. *Cancer Res.*, **49**, 4075.

YEH, G.C., OCCHIPINTI, S.J., COWAN, K.H., CHABNER, B.A. & MYERS, C.E. (1987). Adriamycin resistance in human tumor cells associated with marked alterations in the regulation of the hexose monophosphate shunt and its response to oxidant stress. *Cancer Res.*, **47**, 5994.

ZIJLSTRA, J.G., DE VRIES, E.G.E. & MULDER, N.H. (1987*a*). Multifactorial drug resistance in an adriamycin-resistant human small cell lung carcinoma cell line. *Cancer Res.*, **47**, 1780.

ZIJLSTRA, J.G., DE VRIES, E.G.E., MUSKIET, F.A.J., MARTINI, I.A., TIMMER-BOSSCHA, H. & MULDER, N.H. (1987*b*). Influence of docosahexaenoic acid *in vitro* on intracellular adriamycin concentrations in lymphocytes and human adriamycin-sensitive and -resistant small cell lung cancer cell lines, and on cytotoxicity in the tumor cell lines. *Int. J. Cancer*, **40**, 850.